# Monitoring of SARS-CoV-2 Infection in Ragusa Area: Next Generation Sequencing and Serological Analysis

**DOI:** 10.3390/ijms24054742

**Published:** 2023-03-01

**Authors:** Maria Denaro, Elisa Ferro, Giuseppe Barrano, Salvatore Meli, Mariangela Busacca, Damiano Corallo, Alessia Capici, Alessandra Zisa, Luana Cucuzza, Sandra Gradante, Marialuisa Occhipinti, Paola Santalucia, Raffaele Elia, Angelo Aliquò, Daniele Tibullo, Carmelo Fidone, Vincenzo Bramanti

**Affiliations:** 1 U.O.C. Laboratory Analysis, ASP Ragusa, 97100 Ragusa, Italy; 2 U.O.C. Informatic and Digital Transition Services, ASP Ragusa, 97100 Ragusa, Italy; 3 Top Management Team of ASP Ragusa, 97100 Ragusa, Italy; 4 Department of Biomedical and Biotechnological Sciences, Section of Biochemistry, University of Catania, 95123 Catania, Italy

**Keywords:** COVID-19, SARS-CoV-2, immunoglobulins anti-N IgG, immunoglobulins anti-S1 IgG, immunoglobulins anti-S2 IgG, immunoglobulins anti-RBD IgG, NGS

## Abstract

The coronavirus disease 19 (COVID-19) post pandemic evolution is correlated to the development of new variants. Viral genomic and immune response monitoring are fundamental to the surveillance of severe acute respiratory syndrome coronavirus 2 (SARS-CoV-2) infection. Since 1 January to 31 July 2022, we monitored the SARS-CoV-2 variants trend in Ragusa area sequencing n.600 samples by next generation sequencing (NGS) technology: n.300 were healthcare workers (HCWs) of ASP Ragusa. The evaluation of anti-Nucleocapside (N), receptor-binding domain (RBD), the two subunit of S protein (S1 and S2) IgG levels in 300 exposed vs. 300 unexposed HCWs to SARS-CoV-2 was performed. Differences in immune response and clinical symptoms related to the different variants were investigated. The SARS-CoV-2 variants trend in Ragusa area and in Sicily region were comparable. BA.1 and BA.2 were the most representative variants, whereas the diffusion of BA.3 and BA.4 affected some places of the region. Although no correlation was found between variants and clinical manifestations, anti-N and anti-S2 levels were positively correlated with an increase in the symptoms number. SARS-CoV-2 infection induced a statistically significant enhancement in antibody titers compared to that produced by SARS-CoV-2 vaccine administration. In post-pandemic period, the evaluation of anti-N IgG could be used as an early marker to identify asymptomatic subjects.

## 1. Introduction

SARS-CoV-2 causes a respiratory disease called COVID-19. The COVID-19 disease has caused a significant increase in hospitalizations for pneumonia with multiorgan disease compromising public health and medical services around the world [1].

Furthermore, the introduction of vaccines against SARS-CoV-2 changed the course of the pandemic. In fact, the recent development of vaccines was considered a powerful measure to save lives and minimize the impact on health, social systems, and global economics [2].

In Italy, the COVID-19 vaccination program started on 27 December 2020 for the healthcare workers (HCWs) with the BNT162b2 COVID-19 mRNA vaccine (Pfizer, New York, USA and BioNTech, Mainz, Germany).

The vaccine, administered in two doses 21 days apart, generally has mild side-effects and has been shown to have 95% efficacy in preventing COVID-19 from seven days to two months after the second dose [3], whereas the efficacy decreases to 84% from four to six months after the second dose [4].

It is well known that SARS-CoV-2 genome mutations influence the efficacy of the immune response induced by vaccination.

Since the beginning of the COVID-19 pandemic, numerous mutations of SARS-CoV-2 have been identified. Periodic viral genomic sequencing helps to detect new genetic variants circulating in communities. An updated version of the SARS-CoV-2 phylogenetic tree is shared on GISAID platform (Global Initiative on Sharing Avian Influenza Data). A variant is recognized as a Variant of Concern (VOC) or Variant of Interest (VOI) by the World Health Organization (WHO). A VOC is a variant that presents many malignant changes and demonstrates an enhanced risk of transmissibility, virulence, or changes in clinical disease presentation, impairing the efficacy of diagnostic, preventive, and therapeutic options [5].

VOCs include B.1.1.7 (Alpha), B.1.351 (Beta), P.1 (Gamma), and B1.617.2 (Delta), as well as B.1.1.529 (Omicron). The Omicron variant was the most highly mutated strain compared to the other VOCs, which include BA.1, BA.2, BA.3, BA.4, BA.5, and their respective lineages. Some studies showed a marked reduction of the neutralizing activity of immunity induced by the vaccine against the Omicron variants, especially BA.4 and BA.5 [6].

It is well known that the genome of SARS-CoV-2 (~30 kb) encodes 16 non-structural proteins (NSPs) and 4 main structural proteins, including spike (S), envelope (E), core membrane (M), nucleocapsid (N), and other accessory proteins. 

SARS-CoV-2 neutralizing antibody (nAb) titers have been reported following infection, and these change depending on the length of time from the infection and the severity of the disease [7,8,9]. Further knowledge on the magnitude, timing, and longevity of nAb responses following SARS-CoV-2 infection is vital for understanding the role that nAbs might play in disease clearance and protection from reinfection (also called renewed or second wave infections) or disease [10].

Serum nAbs appear after SARS-CoV-2 infection, typically within 1–3 weeks [11,12] and after vaccination. In the literature, it is known that Ab responses to other human coronaviruses wane over time [13,14,15].

In addition, serological testing helps to estimate the proportion of the population that has been vaccinated or exposed to SARS-CoV-2.

The SARS-CoV-2 antibodies target the two subunit S1 and S2, RBD, and N. Immunoglobulins G targeting N proteins (anti-N IgG) are detectable in the serum of infected patients, whereas IgG targeting S1 subunit protein (anti-S1 IgG), S2 subunit protein (anti-S2 IgG), and RBD (anti-RBD IgG) are detectable in the serum of infected or vaccinated patients.

In the post-pandemic period, it is important to monitor the SARS-CoV-2 variants trend and the evaluation of immune response, both to natural infection and vaccination.

The detection of anti-N IgG could discriminate cases with a history of SARS-CoV-2 infection from cases without a history of infection. Detection of SARS-CoV-2 specific antibodies, especially anti-N IgG, could be an important complementary method not only for the diagnosis of COVID-19, but also for a more accurate evaluation of a population exposed to SARS-CoV-2.

The aim of our study was to provide a monitoring of SARS-CoV-2 infection evolution in Ragusa area since 1 January to 31 July 2022 evaluating: COVID-19 positivity, hospitalization, and death rate; the SARS-CoV-2 variants trend; immune responses to SARS-CoV-2 infection and/or vaccination; and immune responses and clinical symptoms according to the different variants. The preliminary data of this study will be utilized to create an adequate model for monitoring SARS-CoV-2 infection in the post-pandemic period.

Furthermore, the usefulness of anti-N IgG as a potential marker to estimate the exposed subjects to SARS-CoV-2 was evaluated.

## 2. Results

In Table 1, we summarized all data collected: the SARS-CoV-2 infection rate diagnosis, positive patients, and the hospitalization and death rates of subjects in the Ragusa area since 1 January 2022 to 31 July 2022.

### 2.1. SARS-CoV-2 Variants Trend in Ragusa Area and Sicily Region in the Period of January–July 2022

A laboratory analysis of Giovanni Paolo II Hospital in Ragusa is one of the main centers for SARS-CoV-2 sequencing and variant research of the Sicily region. Since 1 January 2022 to 31 July 2022, we sequenced with NGS technology n.600 positive nasopharyngeal swabs to characterize SARS-CoV-2 variants.

In order to compare our data with the regional variants trend of SARS-CoV-2, we also reported data obtained by other sequencing centers for SARS-CoV-2 of Sicily region (IRIDA-ARIES platform/software of the Italian National Institute of Health). In particular, during the period taken into account, in all centers for SARS-CoV-2 sequencing of our region, we sequenced n.10367 nasopharyngeal swabs positive for SARS-CoV-2 infection.

**Figure 1:**
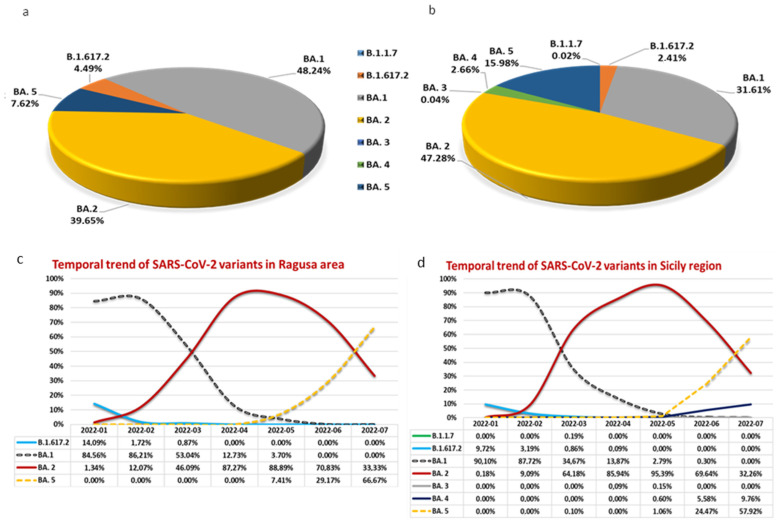
Variants percentages in Ragusa area (**a**) and Sicily region (**b**) and temporal trend with percentage of SARS-CoV-2 variants since 1 January to 31 July 2022 in Ragusa area (**c**) and Sicily region (**d**).

In the Ragusa area, we identified n.4 SARS-CoV-2 variants: B.1.617.2 (4.49%), Omicron BA.1 (48.24%), Omicron BA.2 (39.65%), and Omicron BA.5 (7.62%) (Figure 1a). In the rest of Sicily, Omicron BA.3 variant was also detected with a very low percentage (0.09% in April and 0.15% in May), and Omicron BA.4 variant was detected with a total percentage of only 2.66% (Figure 1b).

As reported in Figure 1, the most representative SARS-CoV-2 variant in our area was Omicron BA.1 (48.24%), whereas in the rest of Sicily region, it was the type Omicron BA.2 (47.28%).

The temporal trend with the respective percentages of the detected SARS-CoV-2 variants in our population, with respect to the Sicily region data, are reported in Figure 1c,d, respectively.

As shown in Figure 1c,d, the trend of SARS-CoV-2 variants in our area with respect to the Sicily region trend was superimposable.

In the Ragusa area, the B.1.617.2 (Delta) variant was detected since January 2022 with a percentage of 14.09% to March 2022 with a percentage of 0.87%. However, during these months, the dominant variant was the Omicron BA.1. Omicron BA.1 and Omicron BA.2, the two variants detected with the highest percentage in our area (48.24% and 39.65%, respectively), coexisted since January to May, although with a reverse trend. During the first month, 84.56% of sequenced samples were Omicron BA.1, whereas 1.34% were the Omicron BA.2 variant. In April and May, the predominant variant was Omicron BA.2 with 88.89% compared to 3.70% of Omicron BA.1 in May. Since May, Omicron BA.5 was detected, becoming the predominant variant in July (66.67%). In our area, the Omicron BA.3 and the Omicron BA.4 variants were not detected in any sequenced nasopharyngeal swabs.

### 2.2. Antibody Response Induced by SARS-CoV-2 Infection

We evaluated the immune response in a cohort of n.600 vaccinated HCWs of ASP Ragusa: n.300 subjects were positive for SARS-CoV-2 infection, whereas the remaining n.300 subjects declared that they had never acquired the SARS-CoV-2 infection. All subjects received n.3 doses of SARS-CoV-2 vaccine: the 1st dose from December 2020 to January 2021; 2nd dose from February to March 2021, and 3rd dose from October 2021 to November 2021.

The evaluation of the immune response to SARS-CoV-2 infection was carried out by blood serum concentration of n.4 immunoglobulins G: anti-N IgG, anti-RBD IgG, anti-S1 IgG, and anti-S2 IgG.

As expected, anti-RBD IgG, anti-S1 IgG, and anti-S2 IgG levels resulted positive both in exposed and unexposed SARS-CoV-2 infection groups. However, we found that the mean of anti-N IgG resulted also positive (29 BAU/mL) in the unexposed to SARS-CoV-2 group (Table 2).

Indeed, among the n.300 HCWs who declared that they never had SARS-CoV-2 infection, n.63 subjects (21%) were seropositive for anti-N antibodies, showing an asymptomatic infection. Consequently, we also performed statistical analysis excluding these cases from the unexposed group.

In Table 2, it was reported that HCWs exposed to SARS-CoV-2 infection were significantly younger than unexposed (*p* = 0.02), conversely no significant difference was observed for sex.

**Figure 2:**
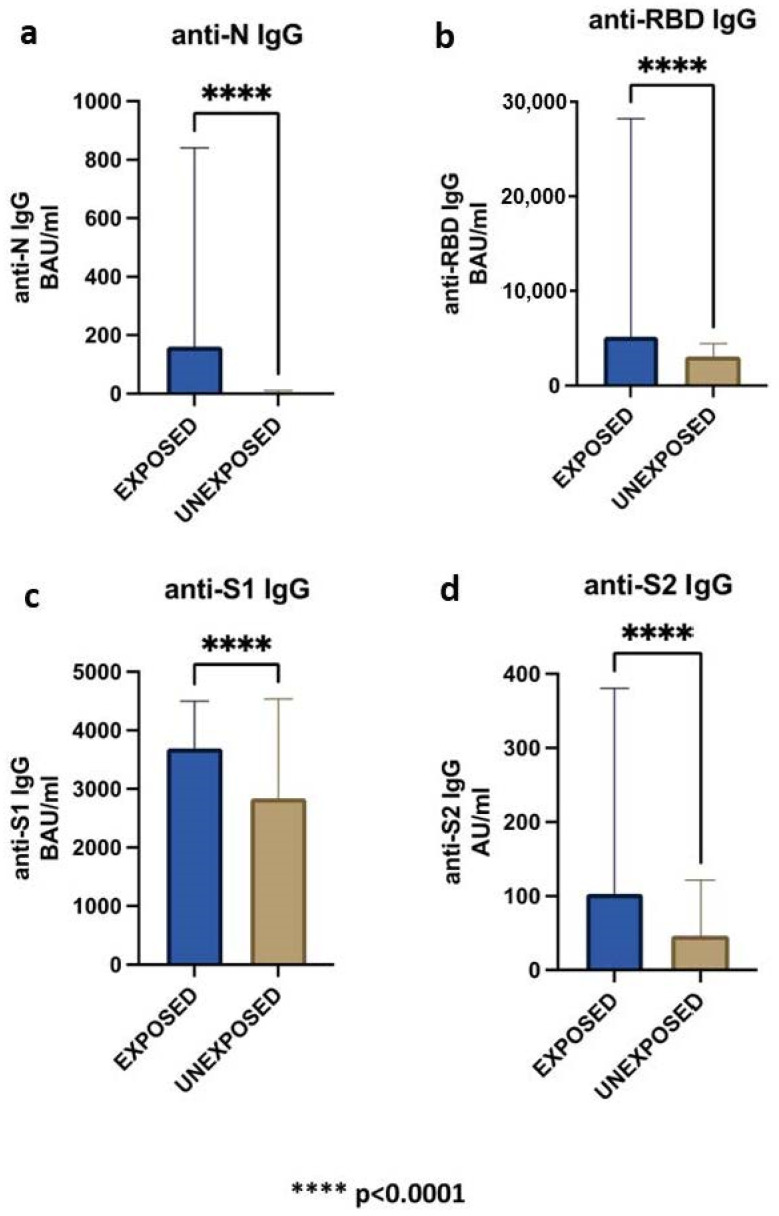
Comparison between exsposed and unexsposed HCWs groups for: anti-N IgG (**a**), anti-RBD IgG (**b**), anti-S1 IgG (**c**) and anti-S2 IgG (**d**).

As expected, all the HCWs were positive for total IgG anti-SARS-CoV-2 and the levels of anti-N IgG were significantly higher in the exposed compared to unexposed group (Table 2, *p* < 0.0001; Figure 2a).

Although the period between the last administration of SARS-CoV-2 vaccine dose and the serological analysis was significantly longer in the exposed than unexposed group (Table 2, *p* = 0.01), and the anti-RBD, anti-S1, and anti-S2 IgG levels were also significantly higher in the exposed group (*p* < 0.0001; Figure 2b–d).

### 2.3. Trend of the Antibody Levels during the Time, Clinical Symptoms, and Presence of SARS-CoV-2 Variants in the Exposed Group

In the SARS-CoV-2 exposed group, the trend of the antibody levels was evaluated considering three characteristics: (1) the interval time between serological analysis and SARS-CoV-2 infection or administration of vaccine dose; (2) clinical symptoms; and (3) the presence of SARS-CoV-2 variants.

Evaluating the trend of the antibody levels during the time from SARS-CoV-2 infection to serological analysis, we found a significant negative correlation for anti-N, anti-RBD, anti-S1, and anti-S2 with R = −6.156 and *p* < 0.00001, R = −4.308 and *p* < 0.0000, R = −6.706 and *p* < 0.00001, and R = −4.661 and *p* < 0.00001, respectively.

In exposed subjects to SARS-CoV-2, a significant correlation was also found for anti-RBD and anti-S2 antibodies levels with the interval time from the last SARS-CoV-2 vaccine dose to the serological analysis (R = −2.536; *p* = 0.011, R = −2.049; *p* = 0.041, respectively). This correlation was not found in the unexposed group.

**Figure 3:**
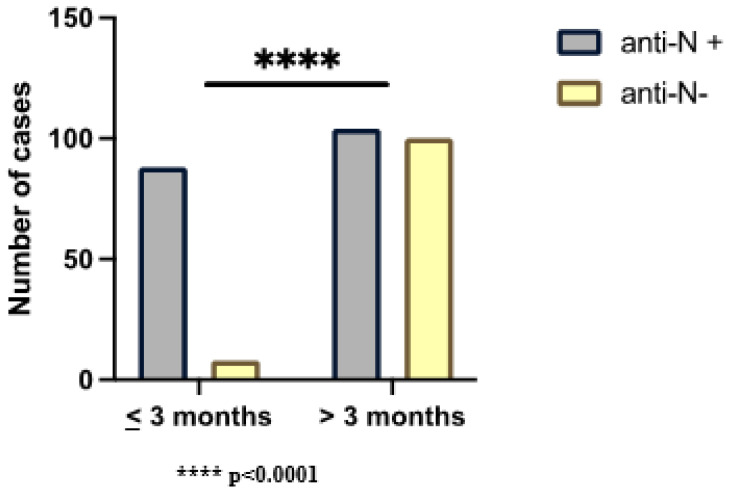
Number of cases seropositive or seronegative for anti-N IgG in relation to SARS-CoV-2 infection in the last 3 months and over 3 months.

In order to evaluate the presence of anti-N IgG during the time (the only antibodies correlated to the natural infection of SARS-CoV-2), we stratified the SARS-CoV-2 exposed group for the period between the infection and serological test: ≤3 months (n.96) and >3 months (n.204). We found a significant statistical correlation (*p* < 0.001) between seropositivity to anti-N IgG, and the presence of SARS-CoV-2 infection occurred ≤3 months. We observed that 92% of infected subjects were seropositive for anti-N IgG ≤ 3 months; in >3 months since infection, 49% of the cases waned in the same antibodies (Figure 3).

In addition, we evaluated cases for anti-S1 and S2 IgG ≤ 6 months and > 6 months since infection, finding that all the analyzed subjects were seropositive both for anti-S1 and anti-S2 IgG ≤ 6 months from infection. Only five cases resulted seronegative for anti-S2 IgG > 6 months from infection.

In order to study and correlate the clinical signs induced by SARS-CoV-2 and also the immune response, we evaluated the symptoms of the patients taken in account. Twenty-one (7%) out of n.300 subjects have been asymptomatic during SARS-CoV-2 infection; the remaining n.279 (93%) showed some specific symptoms. The most frequent clinical manifestations were sore throat (61%), fever (56%), muscle pain (50%), headache (48%) and cough (46%). Other less frequent symptoms were asthenia (12%), loss of smell (5%), loss of taste (4%), vomit (2%), cold (2%), rhinitis (2%), dyspnea (1%), drowsiness (1%), tachycardia (1%), pneumonia (1%), vertigo (1%).

In addition, in order to quantify the severity of clinical symptoms, we classified the subjects as follows: n.1 subjects with only one symptom; n.2 subjects with two symptoms, etc., up to finally n.6 subjects having ≥6 symptoms.

We found no significant difference comparing the stratified groups for variants and symptoms number; however, we found significant direct relationships between an increased number of symptoms and the titer of both anti-N IgG (R = 0.132; *p* = 0.022) and anti-S2 IgG (R = 0.117; *p* = 0.043).

## 3. Discussion

The COVID-19 pandemic caused by SARS-CoV-2 started at the end of December 2019 and is still in evolution. In just over three years, SARS-CoV-2 acquired numerous mutations, giving rise to numerous functional variants with variable degrees of infectivity and lethality. In order to supervise the development and dynamics of SARS-CoV-2 infection, is increasingly important guarantee the “structural” viral genomic surveillance and the host immune response both to natural infection and to vaccination. Next-generation sequencing (NGS) is the gold standard for characterization of SARS-CoV-2 mutations and variant identification [16].

In this paper we studied SARS-CoV-2 infection evolution in Ragusa area in the period: 1 January 2022–31 July 2022. We found that the local scenario in our area reproduced the SARS-CoV-2 infection dynamics of the rest of the Sicily region, although with some exceptions related to Omicron BA.3 and Omicron BA.4 variants presence, leading to the conclusion that the diffusion of the Omicron BA.3 and Omicron BA.4 has affected only some areas of the Sicily region. Omicron BA.1 and Omicron BA.2 were the most representative variants in the first 6 months. Indeed, since July 2022, the Omicron BA.5 variant was the most representative.

According to the regional report Bollettino n.43 of the Sicily region (27 July 2022), since 1 January 2022, there was a significant increase in infected subjects, probably due not only to the easing of mitigation measures, but also to the simultaneous spread of the Omicron variants. In fact, it is well known that the mutations acquired by this variant alter the conformation of RBD, making Omicron able to bind ACE2 better than other variants. In this way, Omicron was characterized by a more significant transmissibility and infectivity [17,18].

In the present study, we tested the sera of n.600 vaccinated HCWs in order to evaluate the SARS-CoV-2 IgG antibody levels. The anti-RBD, anti-S1, and anti-S2 IgG levels were significantly higher in subjects exposed to SARS-CoV-2 infection compared to the unexposed one. These data demonstrated that the SARS-CoV-2 infection induces an immune response with the production of antibodies significantly higher than the antibodies induced by only SARS-CoV-2 vaccine administration.

In the literature, it is known that Ab responses to other human coronaviruses wane over time [12,13,14,15].

We evaluated the trend of the antibody levels and our findings confirmed the literature reports. In the SARS-CoV-2 exposed group, a significant negative correlation between SARS-CoV-2 infection and serological analysis was found for anti-N, anti-RBD, anti-S1, and anti-S2 levels.

Moreover, Long et al. observed that asymptomatic individuals had a weaker immune response to SARS-CoV-2 infection, whereas higher titers and a longer durability of antibodies was observed in symptomatic infections [19]. In addition, it is reported that in the early convalescent phase, 40% of asymptomatic individuals became negative for IgG with respect to 13% of the symptomatic group [19]. Finally, Huang AT et al. concluded that antibody titers remained detectable longer after more severe illness, whereas a more rapid decrement after asymptomatic infection was observed [20].

In our population, we found a significant positive trend between an increased number of symptoms and anti-N IgG and anti-S2 IgG levels.

A small proportion of Omicron infections were consistent with pneumonia, and the majority presented with symptoms of upper respiratory tract infection [21,22]. According to these data in our population, only 1% of the population was affected by pneumonia, whereas the other clinical manifestations included a sore throat (61%), fever (56%), muscle pain (50%), headache (48%), and cough (46%).

As expected, a statistically significant association between SARS-CoV-2 infection and seropositivity to anti-N IgG was found. However, 21% of unexposed subjects to SARS-CoV-2 were positive for anti-N IgG. Most likely, these cases had an infection never diagnosed with a molecular test during the acute phase because it was asymptomatic or with mild symptoms. These data demonstrated that the detection of anti-N IgG could discriminate between exposed with respect to unexposed subjects to SARS-CoV-2 for a more accurate evaluation of infected population.

In a study conducted on 3276 UK healthcare, HCWs was found that anti-S IgG levels remained detected after a positive result at 180 days, whereas for anti-N IgG, the mean estimated half-life was 85 days [23].

Within three months from SARS-CoV-2 infection, 92% of exposed subjects was positive for anti-N IgG; over three months from the infection, 49% of the exposed subjects were negative for the same antibodies.

The results obtained showed that approximately half of the cases with a history for COVID-19 were seronegative for anti-N IgG further than three months from infection. These findings suggest that anti-N IgG could be used as a marker of early infection if tested within n.3 months from the COVID-19 diagnosis. On the contrary, the longer duration of anti-N IgG observed in 51% of cases was probably due to other factors that need to be investigated.

## 4. Materials and Methods

### 4.1. Study Design and Participants

We performed a retrospective study collecting data related to SARS-CoV-2 infection positivity, hospitalization, and death rate of subjects belonging to Ragusa area since 1 January 2022 to 31 July 2022.

Six hundred positive nasopharyngeal swabs were selected and sequenced to monitoring the SARS-CoV-2 variants trend in our area. The variants trend of Ragusa area was compared to that of the Sicily region, downloading from ISS Platform I-Co-Gen the sequencing results of the region.

In order to evaluate the effect of SARS-CoV-2 natural infection and/or vaccination on immune response, the HCWs of ASP Ragusa were invited to participate in this study, which compiled an anonymous questionnaire about age, SARS-CoV-2 infection status, data of SARS-CoV-2 infection, symptoms, and vaccination status (Supplementary Materials). Informed consent was obtained from all subjects involved in the study.

HCWs represented a very interesting population for our study for their high risk of exposition to SARS-CoV-2 infected patients and for their regularly vaccination status.

Six hundred HCWs were involved in this study: n.300 HCWs declared that they never contracted the SARS-CoV-2 infection; n.300 were positive for SARS-CoV-2 infection. These last positive ones were characterized for variant type and included in the sequenced samples as previously reported.

The evaluation of immune response to SARS-CoV-2 infection was carried out in all HCWs involved in the study evaluating the serum levels of anti-N, anti-RBD, anti-S1, and anti-S2 IgG.

In the SARS-CoV-2 exposed group, the trend of SARS-CoV-2 IgG levels was evaluated considering: the interval time between serological analysis and SARS-CoV-2 infection and/or administration of vaccine dose, clinical symptoms, and SARS-CoV-2 variant.

Finally, we evaluated the usefulness of anti-N IgG as potential marker to estimate the exposure to SARS-CoV-2.

### 4.2. SARS-CoV-2 Genome: Extraction and Sequencing

The SARS-CoV-2 genome was extracted from nasopharyngeal swabs that resulted positive from RT-PCR with Ct values < 30. The extraction was performed using MagCore^®^ Viral Nucleic Acid Extraction Kit (RBC Bioscience, New Taipei City, Taiwan).

Viral whole genome SARS-CoV-2 sequencing was performed using QIAseq DIRECT SARS-CoV-2 protocol (Qiagen, Hilden, Germany). After the cDNA synthesis, according to the manufacturer’s protocol, two library pools with different couples of primers were set up for each sample. The pools were mixed into a single tube and successively purified using a QIAseq Bead cleanup (Qiagen, Hilden, Germany). Then, samples were indexed with unique dual indexes.

Concentrations of single pools were established with Qubit 4 Fluorometer and Qubit dsDNA HS (high sensitivity) Assay Kit (Thermo Fisher Scientific, Wilmington, DE, USA). Each pool was diluted with RNAse free-water to obtain the concentration according to the protocol and pooled. The sequencing was performed using MiSeqDx system (Illumina San Diego, CA, USA) with a V3 cartridge in 149 bp paired-end reads.

Informatic variant calling, data quality, and consensus file were executed with Glc Genomics workbench 22.0. The lineage analysis and classification was made with Pangolin COVID-19 lineage assigner software v 4.1.3. All sequences and variant calls were uploaded to ISS Platform I-Co-Gen and GISAID [24,25].

### 4.3. SARS-CoV-2 IgG Panel Test

SARS-CoV-2 IgG panel test (BioRad, Hercules, CA, USA) is a multiplex assay to detect the following anti-SARS-CoV-2 IgG: receptor-binding domain (RBD), spike 1 (S1), spike 2 (S2), and nucleocapsid (N) antibodies. Unlike the others antibodies, only anti-N IgG are related to SARS-CoV-2 infection. Therefore, the presence of the anti-N IgG allowed us to distinguish antibody response from natural infection and vaccination.

This panel test was performed using the Bioplex 2200 (Bio-Rad, USA). The assay employed fluoromagnetic dyed beads coated with RBD, protein S1 subunit, protein S2 subunit, and protein N.

The panel also provided composite qualitative positive/negative result of total IgG. The results for the different SARS-CoV-2 IgG were quantitative and were reported in BAU/mL for anti-N, anti-RBD, and anti-S1, as well as semi-quantitative results in AU/mL for anti-S2.

According to BioPlex 2200 SARS-CoV-2 IgG Panel protocol: anti-N IgG are negative for values < 23 BAU/mL and positive for values > 24 BAU/mL; anti-RBD IgG are negative for values < 12 BAU/mL and positive for values > 13 BAU/mL; anti-S1 IgG are negative for values < 21 BAU/mL and positive for values > 22 BAU/mL; and anti-S2 IgG are negative for values < 10 AU/mL and positive for values > 10 AU/mL.

### 4.4. Bioplex 2200 SARS-CoV-2 IgG: Principle of the Procedure

The assay employed fluoromagnetic dyed beads coated with RBD, protein S1 subunit, protein S2 subunit, and N protein. In addition, an Internal Standard Bead (ISB) to verify the detector response and a Serum Verification Bead (SVB) to verify the presence of sample in the reaction were present in each reaction mixture. The system was calibrated using a set of six distinct calibrators [26], and quality control was performed using a control set.

The BioPlex 2200 System combined and incubated at 37 °C a mixture composed of an aliquot of serum sample, diluent, and beads reagent. Murine monoclonal anti-human IgG conjugated to phycoerythrin (PE) was added to the dyed beads after a wash cycle. This mixture was incubated at 37 °C. Another wash cycle removed the excess of conjugate. The beads were re-suspended in sheath fluid and conducted to a detector. Each bead had a different fluorescence, and the amount of antibody captured by the antigen was determined by the fluorescence of the attached PE. Raw data were calculated in relative fluorescence intensity (RFI). All calculations necessary to interpret the results were performed automatically by the BioPlex 2200 System Software (Bio-Rad, USA). The qualitative results were reported as negative or positive.

### 4.5. Statistical Analysis

Analyses were performed using GraphPad Prism 9.1.2 and the Statistic program (version 10.0). In order to assess the distribution patterns, Lilliefor and Shapiro–Wilk normality tests were used. Continuous variables were expressed as mean ± standard deviation (SD). The non-parametric Mann-Whitney and Spearman tests were used. Categorical data were expressed as number and percentage and analyzed using the Chi-squared Test. A *p*-value < 0.05 was considered statistically significant.

## 5. Conclusions

The COVID-19 post pandemic evolution is inevitably correlated to an incessant development of new variants. The viral genomic and the immune response monitoring represent the main factors related to the surveillance of SARS-CoV-2 infection.

We demonstrated that the SARS-CoV-2 infection variants trend of our area reproduce the same ones of the rest of the Sicily region. Omicron BA.1 and BA.2 have been the most representative variants, both in our area and in the rest of the region, whereas the diffusion of the Omicron BA.3 and Omicron BA.4 occurred only in some parts of the region.

Our findings demonstrated that the SARS-CoV-2 infection induced an enhancement of antibody titers than ones produced by only SARS-CoV-2 vaccine administration.

Considering the temporal dynamics, a significant negative correlation was found for anti-N, anti-RBD, anti-S1, and anti-S2 IgG between SARS-CoV-2 infection and serological analysis.

Instead, only anti-N IgG and anti-S2 IgG levels were also positively correlated with the increase in the number of symptoms.

On the other hand, almost all exposed subjects were positive for anti-N IgG within three months of the infection. In the post-pandemic period, the evaluation of anti-N IgG could be used as an early marker to identify asymptomatic subjects.

It is well known that retrospective studies, such as the current one, have some limitations. In fact, they measure events that occurred before the study design. Despite this, our findings highlight the importance of a monitoring in post-pandemic period. Prospective and multicenter studies are required not only to confirm the obtained results but also to investigate the ability of SARS-CoV-2 variants to stimulate or escape the immune system. In the future, the monitoring of the immune response over time would also be recommended.

## Figures and Tables

**Table 1:** Positivity, hospitalization and death rate data of Ragusa area population.

Tested Nasopharyngeal Swabs	Positive Nasopharyngeal Swabs	Positive Patients Hospitalized	Died Patients
500,475	93,735 (18.72%)	834 (0.88%) 49 (0.05%) intensive care unit	213 (0.22%)

**Table 2:** Questionnaire and SARS-CoV-2 IgG Panel data with comparison between exposed and unexposed subjects.

Data Media ± DS	Total Vaccinated Workers n.600	Exposed n.300	Unexposed n.300	Unexposed * n.237	*p*-Value
**AGE (years)**	53 ± 10	52 ± 11	54 ± 10	54 ± 10	0.02 (0.02)
**Sex** **Number (%)**	Females 393 (65.50%) Males 207 (35.50%)	Females 205 (68.33%) Males 95 (3267%)	Females 188 (62.66%) Males 112 (37.33%)	Females 149 (62.86%) Males 88 (37.14%)	0.17 (0.11)
**Total IgG (%Positive)**	100% Positive	100% Positives	100% Positives	100% Positives	
**Anti-N IgG**	75 ± 196 BAU/mL	121 ± 257 BAU/mL	29 ± 88 BAU/mL	4 ± 5 BAU/mL	<0.0001 (<0.0001)
**Anti-RBD IgG**	3537 ± 1053 BAU/mL	3836 ± 678 BAU/mL	3261 ± 1246 BAU/mL	3074 ± 1341 BAU/mL	<0.0001 (<0.0001)
**Anti-S1 IgG**	3349 ± 1304 BAU/mL	3684 ± 815 BAU/mL	3035 ± 1573 BAU/mL	2838 ± 1699 BAU/mL	<0.0001 (<0.0001)
**Anti-S2 IgG**	61 ± 74 AU/mL	70 ± 77 AU/mL	51 ± 71 AU/mL	47 ± 75 AU/mL	<0.0001 (<0.0001)
**Months from** **last vaccine dose to serological test**	9 ± 2 Months	9 ± 3 Months	8 ± 2 Months	8 ± 2 Months	0.001 (0.01)
**Months from** **infection to serological test**	-------------	6 ± 5 Months	---------------	-----------	

* Exposed group are compared to unexposed one after exclusion of asymptomatic subjects with positive IgG anti-N values and relatives *p*-values are reported in brackets.

## Data Availability

Not applicable.

## Supplementary Materials

The supporting information can be downloaded at: https://www.mdpi.com/article/10.3390/ijms24054742/s1.
