# Supplementary material for: Monitoring of SARS-CoV-2 Infection in Ragusa Area: Next Generation Sequencing and Serological Analysis
Source: Int J Mol Sci. 2023 Mar 1;24(5):4742. doi: 10.3390/ijms24054742 (PMC10003428; doi:10.3390/ijms24054742)
Supplement: Supplementary file 1 [file ijms-24-04742-s001.zip › ijms-2210586-supplementary.pdf]

ASP RAGUSA
AZIENDA SANITARIA PROVINCIALE

MONITORING OF SARS-COV-2 IMMUNE RESPONSE PROJECT

The anti-SARS-CoV-2 IgG serological test evaluates total anti-SARS-CoV-2 IgG and discriminates the subclasses of IgG induced by SARS-CoV-2 natural infection and/or vaccination (anti-RBD, anti-S1 and anti-S2) or by only natural infection (anti-N).
All HCWs of ASP Ragusa are invited to participate at this project voluntary, writing following anonymous questionnaire.

Date of birth______________________________________ Code________________________________

Hospital unit and related operating unit____

- Have you ever affected of COVID-19? ☐ Yes ☐ No

- Report the date of <u>positive</u> test ____

- Clinical symptoms related to SARS-CoV-2 infection:

  ☐ None ☐ Fever ☐ Cough ☐ Sore throat

  ☐ Headache ☐ Muscle aches ☐ Other____

- Have you been hospitalized for COVID-19 ☐ Yes ☐ No

- The last SARS-CoV-2 vaccination dose administered ☐ I dose ☐ II dose ☐ III dose

- Date of vaccination dose administered _______________________________________

DATE __________________
